# Reduced stem cell aging in exercised human skeletal muscle is enhanced by ginsenoside Rg1

**DOI:** 10.18632/aging.203176

**Published:** 2021-06-28

**Authors:** Tania Xu Yar Lee, Jinfu Wu, Wei-Horng Jean, Giancarlo Condello, Ahmad Alkhatib, Chao-Chieh Hsieh, Yu-Wen Hsieh, Chih-Yang Huang, Chia-Hua Kuo

**Affiliations:** 1 Laboratory of Exercise Biochemistry, University of Taipei, Taipei City 11153, Taiwan, ROC; 2 Department of Anesthesiology, Far East Memorial Hospital, New Taipei City 220, Taiwan, ROC; 3 School of Health and Life Sciences, Teesside University, Middlesbrough TS1 3BX, England, United Kingdom; 4 Laboratory of Regenerative Medicine in Sports Science, School of Physical Education & Sports Science, South China Normal University, Guangzhou, China; 5 Cardiovascular and Mitochondrial Related Disease Research Center, Hualien Tzu Chi Hospital, Buddhist Tzu Chi Medical Foundation, Hualien 970, Taiwan, ROC; 6 Center of General Education, Buddhist Tzu Chi Medical Foundation, Tzu Chi University of Science and Technology, Hualien 970, Taiwan, ROC; 7 Department of Medical Research, China Medical University Hospital, China Medical University, Taichung 404, Taiwan, ROC; 8 Graduate Institute of Biomedical Sciences, China Medical University, Taichung 404, Taiwan, ROC

**Keywords:** p16^INK4a^, resistance exercise, Rg1, senolytic, cellular senescence, CD4

## Abstract

Background: Stem cell aging, characterized by elevated p16^INK4a^ expression, decreases cell repopulating and self-renewal abilities, which results in elevated inflammation and slow recovery against stress.

Methods: Biopsied muscles were analyzed at baseline and 24 h after squat exercise in 12 trained men (22 ± 2 y). Placebo (PLA) and immunostimulant Rg1 (5 mg) were supplemented 1 h before a squat exercise, using a double-blind counterbalanced crossover design.

Results: Perceived exertion at the end of resistance exercise session was significantly lowered after Rg1 supplementation. Exercise doubled endothelial progenitor cells (EPC) (*p* < 0.001) and decreased p16^INK4a^ mRNA to 50% of baseline (*d* = 0.865, *p* < 0.05) in muscle tissues, despite p16^INK4a+^ cell and beta-galactosidase^+^ (ß-Gal^+^) cell counts being unaltered. Rg1 further lowered p16INK4a mRNA to 35% of baseline with greater effect size than the PLA level (*d* = 1.302, *p* < 0.01) and decreased myeloperoxidase (MPO) mRNA to 39% of baseline (*p* < 0.05). A strong correlation between MPO and p16^INK4a^ expression in muscle tissues was observed (*r* = 0.84, *p* < 0.001).

Conclusion: EPC in skeletal muscle doubled 1 d after an acute bout of resistance exercise. The exercised effects in lowering EPC aging and tissue inflammation were enhanced by immunostimulant Rg1, suggesting the involvement of immune stimulation on EPC rejuvenation.

## INTRODUCTION

Stem cell aging is characterized by increased expression of the cell cycle inhibitor p16^INK4a^ in replicable cells [1]. This inhibitor halts cell regeneration, self-renewal and homing, resulting in stress intolerance and fitness decline at a higher age [2, 3]. Increasing p16^INK4a^ expression during cellular aging is also an intrinsic mechanism to lower mitotic error of aged stem cells [4]. Since most of cells within the human body are short-lived [5], p16^INK4a+^ senescent cells are widely detectable in embryonic [6] and young adult tissues [7–9]. Lymphocyte p16^INK4a^ mRNA increases exponentially with age from 18 to 80 years old [10]. However, p16^INK4a+^ cell count is not different between young and old muscles measured by semi-quantitative immunohistochemical (IHC) analysis [9]. Stem cells express a wide spectrum of p16^INK4a^ mRNA. Therefore, this discrepancy suggests that the level of p16^INK4a^ mRNA is probably a more discernable biomarker of tissue aging than counting p16^INK4a+^ cell number in humans.

The effect of an acute bout of resistance exercise on p16^INK4a^ mRNA in skeletal muscle of trained humans has not been previously reported. Based on IHC analysis, resistance exercise seems to suppress p16^INK4a+^ cell number surrounding myofibers in skeletal muscle of untrained men [7] and physically inactive women [3]. A significant proportion of p16^INK4a+^ cells are colocalized with CD34^+^ endothelial progenitor cells (EPC) adjacent to myofibers [7]. EPC contributes to cell regeneration of both vascular endothelium [11] and myofibers in damaged muscle tissues [12]. Vascular endothelial cells in muscle tissue age rapidly with a lifespan of around two weeks [13]. Therefore, the levels of EPC aging and young EPC availability for cell regeneration directly influence the fitness of muscle tissues. Resistance exercise acutely mobilizes EPC from bone marrow into circulation until 6 h but quickly returns them to baseline within 24 h of recovery [14]. It remains unknown whether EPC will be settled and proliferated in human skeletal muscle tissues during the 24-h recovery period following an acute bout of resistance exercise. Since young stem cells have greater proliferating potential than old stem cells, it would be important to assess whether p16^INK4a^ mRNA can be lowered in young human muscle tissues using a validated resistance exercise protocol for muscle hypertrophy [15].

Cellular senescence in tissues contributes to baseline inflammation [16]. This phenomenon is characterized by increased neutrophil infiltration in tissues, which can be detected by MPO mRNA level in tissue [17]. During inflammation, the phagocytes recognize and eliminate senescent cells to maintain youth of the tissue [18]. However, most of the aforementioned knowledge comes from *in vitro* and animal studies, whilst no evidence is currently available in human skeletal muscle. In light of the paucity of knowledge, this study was undertaken to measure EPC number and p16^INK4a^ mRNA in human skeletal muscle together with neutrophil markers after an acute bout of the resistance exercise that normally generates muscle damage and hypertrophy.

Rg1 is a ginseng-based immunostimulant with the effect of enhancing neutrophil migration in animals [19, 20]. Its potential effect has also been investigated in young men. In particular, Rg1 attenuates satellite cells depletion and increase myogenic gene expression after 1 h aerobic cycling at 70% VO_2max_ [21]. Furthermore, Rg1 enhances the activation of phagocytosis for ß-Gal^+^ cell clearance during high intensity aerobic cycling and increase endurance performance [22]. However, current evidence on the potential functions of Rg1 in human skeletal muscle is not available in response to a resistance exercise aimed for muscle hypertrophy-related immunostimulation. To examine the role of immune stimulation in human skeletal muscle, the Rg1 was supplemented 1 h before resistance exercise. We hypothesized a further decrease in senescent cell markers (p16^INK4a^ mRNA and p16^INK4a+^ EPC) and neutrophil markers (MPO mRNA) in the exercised skeletal muscle after Rg1 supplementation with 24 h post-exercise recovery in humans.

## RESULTS

**Figure 1:**
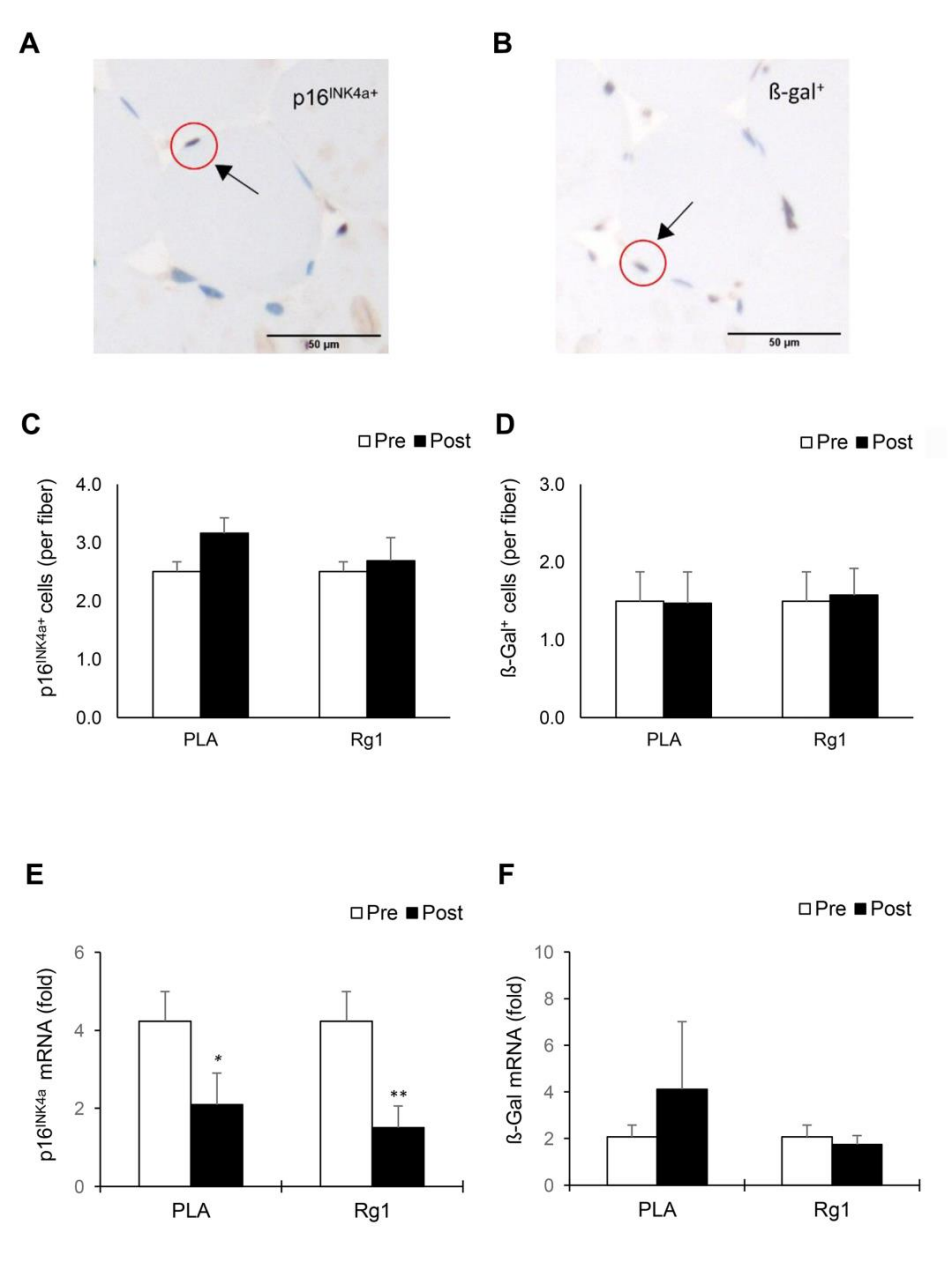
**Biomarkers of cellular senescence in human skeletal muscle 24 h after squat exercise.** The p16^INK4a+^ cells (**A**) and ß-Gal^+^ cells (**B**) are indicated by brown precipitates surrounding myofibers in the immunohistochemical stains of muscle cross-sections. No significant changes in quantity of p16^INK4a+^ cells (**C**) and ß-Gal^+^ cells (**D**) Were observed post exercise during PLA- and Rg1-supplemented trials. Squat exercise decreased p16^INK4a^ mRNA (**E**) While no effect on ß-Gal mRNA (**F**) in vastus lateralis muscle of young men with training experience. ^*^denotes significant difference against Pre, *p* < 0.05; ^**^denotes significant difference against Pre, *p* < 0.01. Abbreviations: Pre: before exercise; Post: 24 h after exercise. ß-Gal^+^: ß-Galactosidase; PLA: Placebo; Rg1: Ginsenoside Rg1.

IHC data (expressed as positive cell number per myofiber) for p16^INK4a+^ cells and ß-Gal^+^ cells of muscle cross-sections are shown in Figure 1. The p16^INK4a+^ cells and ß-Gal^+^ cells were located surrounding myofiber (Figure 1A and 1B) and were unchanged 24 h following resistance exercise under both PLA- and Rg1-trials (Figure 1C and 1D). Squat exercise decreased p16^INK4a^ mRNA of vastus lateralis muscle to 50% (*d* = 0.865, *p* = 0.034) of baseline in the PLA-trial and further decreased to 35% (*d* = 1.302, *p* = 0.007) (Figure 1E) of baseline in the Rg1-trial, respectively (main effect of time, *p* = 0.002). Exercise effect on ß-Gal mRNA was not significant for both PLA- and Rg1-trials.

**Figure 2:**
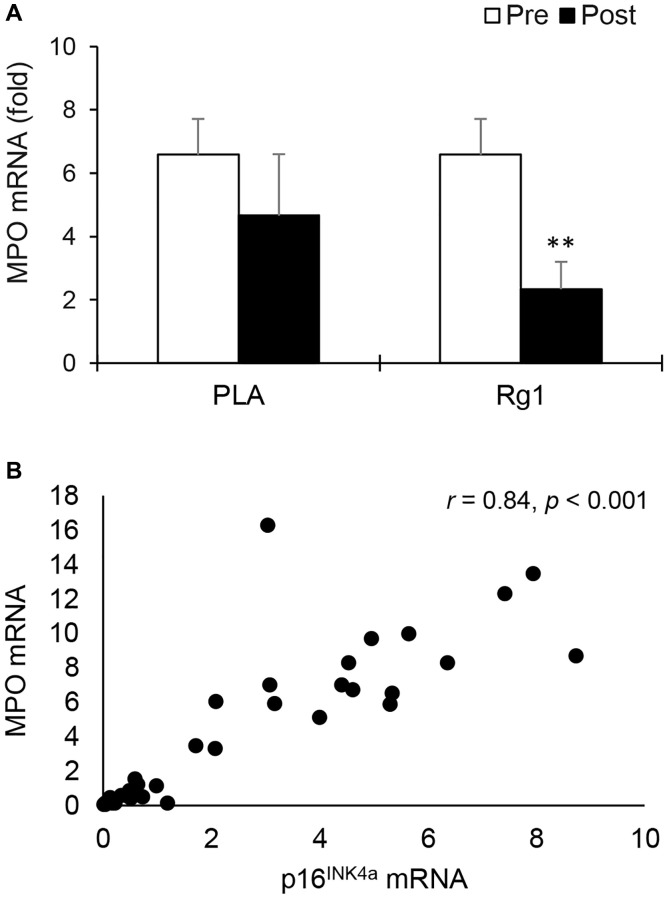
**Myeloperoxidase mRNA in human skeletal muscle 24 h after squat exercise.** Myeloperoxidase mRNA (neutrophil marker) decreased post exercise when immunostimulant Rg1 was supplemented 1 h before exercise (**A**). Myeloperoxidase mRNA is highly correlated with p16^INK4a^ mRNA (**B**) In muscle tissues. ^**^denotes significant difference against Pre, *p* < 0.01. Abbreviations: Pre: before exercise; Post: 24 h after exercise. MPO: Myeloperoxidase; PLA: Placebo; Rg1: Ginsenoside Rg1.

MPO mRNA (neutrophil marker) also decreased 24 h after squat exercise (main effect of time, *p* = 0.026). Pre-exercise Rg1 supplementation induced more significant decreases in MPO mRNA below the PLA level (PLA: *d* = 0.382, *p* = 0.196; Rg1: *d* = 1.330, *p* < 0.01) (Figure 2A). When all muscle tissues were pooled together, a very large correlation was found between MPO mRNA and p16^INK4a^ mRNA (*r* = 0.84, *p* < 0.001) (Figure 2B). A significant correlation was also found between MPO mRNA and ß-Gal mRNA (*r* = 0.56, *p* = 0.001).

**Figure 3:**
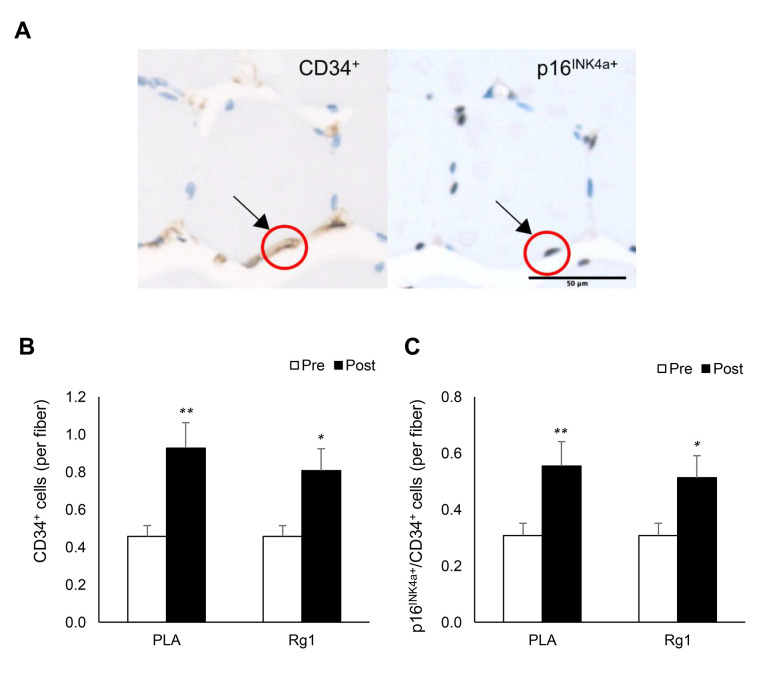
**Endothelial progenitor cell expansion (CD34^+^ cells) in human skeletal muscle 24 h after squat exercise.** The CD34^+^ cells and p16^INK4a+^ cells (**B**) are indicated by brown precipitates surrounding myofibers in immunohistochemical stains of muscle cross-section. (**A**) CD34^+^ cells were nearly doubled post exercise (**C**). Approximately 65% of CD34^+^ cells were colocalized with p16^INK4a+^ cells. This ratio was not much affected after exercise (60%) in the trained men. ^*^denotes significant difference against Pre, *p* < 0.05; ^**^denotes significant difference against Pre, *p* < 0.01. Abbreviations: Pre: before exercise; Post: 24 h after exercise. PLA: Placebo; Rg1: Ginsenoside Rg1.

Some of p16^INK4a+^ cells in the muscle cross-section were colocalized with EPC (CD34^+^ cells) (Figure 3A). EPC in muscle tissue almost doubled 24 h after resistance exercise for both PLA- and Rg1-trials (PLA: *d* = 1.383, *p* < 0.001; Rg1: *d* = 1.095, *p* = 0.015) (Figure 3B). Despite unchanged total p16^INK4a+^ cells (positive cell per fiber) in muscle after exercise, p16^INK4a+^/CD34^+^ cells were selectively elevated during both PLA- and Rg1-trials (PLA: *d* = 1.047, *p* = 0.003; Rg1: *d* = 0.933, *p* = 0.010) (Figure 3C).

**Figure 4:**
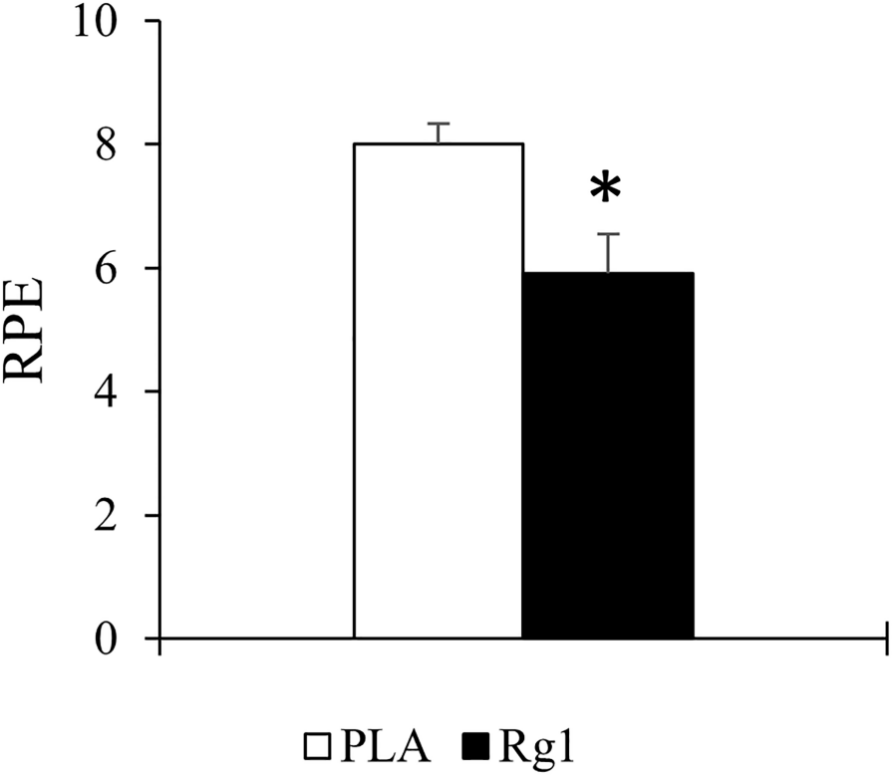
Rating of perceived exertion (RPE) at the end of resistance exercise session was significantly lowered after 1 h after Rg1 supplementation. ^*^Significant difference between PLA and Rg1, *p* < 0.05.

Rg1 decreased subjective perceived effort (RPE) from 8 ± 0.3 AU to 6 ± 0.6 AU at the end of resistance exercise (*d* = 1.195, *p* = 0.007) (Figure 4). Among these trained men, no significant differences were found in the post-exercise muscle soreness measurements (VAS scale at post, 24 h, 48 h, 72 h) between both PLA- and Rg1-trials (*d* = 0.065, *p* = 0.881).

## DISCUSSION

The key findings of the study are 1) a doubled EPC and decreased p16^INK4a^ mRNA in human skeletal muscle 1 day after squat exercise; 2) the exercise-induced EPC rejuvenation and attenuated muscle inflammation were enhanced by pre-exercise Rg1 supplementation. No change in p16^INK4a+^ cell number is probably associated with low levels of p16^INK4a+^ senescent cell number in muscle tissues of men with regular training [3, 7]. It is likely that all replicable cells in the muscle tissue express a wide spectrum of p16^INK4a^ mRNA levels. Therefore, p16^INK4a^ mRNA seems to be a more sensitive biomarker to detect the level of tissue senescence in human skeletal muscle [23], compared with the semi-quantitative IHC analysis of p16^INK4a+^ cell count [9]. Rg1 is a natural immunostimulant [19, 20]. Thus, the greater effect size in the reductions of MPO mRNA and p16^INK4a^ mRNA in the exercised muscle after Rg1 supplementation also suggests an involvement of immune stimulation in lowering cellular senescence and baseline inflammation.

Another novel finding of the study is a very large correlation (*r* = 0.84) between p16^INK4a^ and MPO expression in human muscle tissues. This result supports the notion that cellular senescence in normally tissues attracts neutrophil infiltration to elevate inflammation, suggested by animal and *in vitro* studies [16, 24]. MPO is expressed specifically in neutrophils [17] which infiltrates in muscle tissue during phagocytic phase of inflammation [25]. Low MPO expression found in this study may mirror a reduced demand for neutrophil-mediated phagocytosis after sufficient period of tissue renewal in recovered human muscles [25]. This finding implicates an immunity sparing effect of exercise by rejuvenating muscle tissues. Among the same men, only a 31% common variance was observed between ß-Gal and MPO mRNA, which suggests that ß-Gal expression may not be a sensitive biomarker to reflect the magnitude of cellular senescence of human skeletal muscle.

Exercise induces a brief surge of circulating EPC in 6 h and quickly diminished to pre-exercise baseline within 24 h [14]. In this study, we further demonstrated that EPC is settled and proliferated in human skeletal muscle 24 h after resistance exercise. EPC contributes to fast regeneration of vascular endothelial cells and donates nucleus during myofiber regeneration [12]. Therefore, increasing EPC availability in tissues provides advantage in accelerating repair process for fast replacement of short-lived endothelial cells against daily challenges [26]. This finding of EPC expansion in exercised human skeletal muscle provides a novel mechanism which explains the benefit of resistance exercise on vascular health and skeletal muscle adaptability.

An important finding of this study is the lowered perceived exertion against squat exercise after the Rg1 supplementation (*p* < 0.05). The underlying mechanism remains to be explored. The immediate effect in perceived exertion implicates a central action of Rg1 to facilitate muscle recruitment by the brain [27]. Furthermore, the Rg1 effect appears to influence the actual internal load of the exercise, which may in turn lead to the absence of group differences in some variables in the study.

In terms of the study’s limitations, we are aware that a single time point biopsy might be insufficient to delineate the dynamical changes of p16^INK4a^ expression, which restricts our physiological interpretation of its expression after an acute bout of exercise. Muscle damage is generally followed by a period of phagocytosis and cell renewal. Therefore, further aging and death of challenged cells can occur at earlier biopsy time point during and after exercise. We have recently observed a 21-fold increase in p16^INK4a^ cells in human muscle 3 h post aerobic exercise. Such exercise type creates a much lower muscle damage than resistance exercise, likely promoting further aging but not death of unfit senescent cells [28]. This acute exercise-induced response in p16^INK4a^ cell surge may serve to induce inflammation mechanism for enhancing phagocytosis and cell regeneration to rejuvenate the challenged tissues [24]. We do not know whether the peak of p16^INK4a^ cells surge occurs during the resistance exercise, since it has obviously returned below baseline at 24 h following recovery. Furthermore, whether the resistance exercise-induced reduction in stem cell aging can be accelerated by Rg1 in older individuals requires further investigation.

## CONCLUSIONS

The results of the study suggest that reduced stem cell aging in exercised human skeletal muscle is enhanced by ginsenoside Rg1 supplementation. A strong correlation between p16INK4a and MPO expression in human skeletal muscle provides the first support of cellular senescence-induced inflammation in human tissues. Pre-exercise Rg1 supplementation decreased MPO mRNA and exerts a greater effect size in EPC senescence-lowering effect in exercised muscle tissues following 24 h recovery, suggesting a role for immune stimulation in stem cell rejuvenation.

## MATERIALS AND METHODS

### Ethical approval

The study was approved by the Institutional Review Board of University of Taipei, Taipei, Taiwan (IRB-2017–41). All experimental procedures were conducted in accordance with the Declaration of Helsinki [29]. Participants were given full explanation of the purpose, experimental procedure, and the potential risks of participation. Written informed consent was received prior to the commencement of the study.

### Participants

Participants with cardiovascular, pulmonary, metabolic, bone or joint problem, smoking habit, muscle and joint injuries during the past 6 months were excluded. Eighteen young men were enrolled to participate in this study with 6 dropouts due to time conflict. A total of 12 men (age: 22 ± 2 years; height: 173 ± 4 cm; weight: 72 ± 8 kg) with an experience of weight training (at least 2 years, ≥ 3 times/week) completed this study.

### Study design

To assess the muscle response after an acute bout of squat exercise under PLA- and Rg1-supplemented conditions, a randomized, double-blind placebo-controlled, counter-balanced crossover study design was conducted with a 3-week washout period. Following the 12-h overnight fast, participants were randomized to the PLA- and Rg1-trials in a counter-balanced order and the supplements were orally delivered 1 h before squat exercise. Capsules containing PLA (cornstarch) or ginsenoside Rg1 were orally delivered to participants via a drink (Herbalife Formula One shake, California, USA) 1 h before the squat exercise in the morning. This drink at a protein dose of 0.12 g·kg^-1^·body weight was supplied for the purpose of providing sufficient nitrogen and carbon for enhancing muscle protein synthesis [30]. Participants could not distinguish the difference between PLA and Rg1 supplements during oral delivery. A Rg1 dose of 5 mg was used in this study (NuLiv Science, Inc., Brea, CA, USA), based on a previous report [21].

Rating of Perceived Exertion (RPE) was self-reported immediately after the exercise [31]. Each participant completed the 1 repetition maximum (1-RM) assessment, as a maximum muscle strength assessment, three weeks prior to squat exercise. Muscle biopsies were performed at baseline and 24 h after squat exercise. The baseline muscle biopsy was performed > 10 days before squat exercise. Participants were informed to stop any form of their own training activity for a week before the baseline biopsy and the exercise challenge. Post-exercise muscle biopsy was performed 24 h after the squat exercise (25 h after PLA or Rg1 supplementation) to allow a short recovery.

### Maximum leg strength

Maximum muscle strength was determined as 1-RM based on a previous protocol [15]. After a warm-up exercise consisted of dynamic stretching exercises, participants conducted a specific warm-up included a set of 10 repetitions with 50% of an estimated 1-RM (according to perceived capacity), a set of 5 repetitions with 75% of the estimated 1-RM, and a final set of 1 repetition with 90–95% of the estimated 1-RM. After a 5-min rest period, participants completed 3 to 5 attempts with progressively heavier weights (∼5%), interspersed with 3–5 min rest intervals, until a 1-RM was achieved. Participants were instructed to adopt a shoulder width stance in keeping with their normal squat stance, descend in a controlled manner, avoid bouncing at the bottom position, maintain as near a vertical torso as possible, and feet always flat on the ground.

### Squat exercise

A qualified trainer instructed the exercise protocol for all participants within a weight training room to ensure the consistency of the challenge. Each participant was required to perform a back-squat exercise using a barbell and a power half squat rack. Participants were advised to lower the barbell until their knees reach 90°, by having the gluts touching the adjustable multi-angle gym bench. Each participant completed his own set of warm-up followed by a structured set of 8 repetitions (50% 1-RM) of barbell back squat. The squat exercise consisted of 6 sets of 8 repetitions (70% 1-RM) with a 90-s rest interval between sets. Maximum weight lifted in relation to body weight ranged from 0.97 to 1.38 kg/kg body weight.

### Rates of perceived exertion and pain

RPE was self-reported immediately after the completion of the exercise. Participants reported the RPE by observing a numerical scale, ranging from 1 “rest” to 10 “maximal effort”. The RPE scale was also based on repetitions in reserve (RIR) for resistance exercise [32]. Before (Pre) and after (Post, 24 h, 48 h, 72 h) the exercise protocol, perceived muscle soreness/pain were assessed with the visual analog scale (VAS), using a continuous 10-cm scale anchored by two verbal descriptors labeled from the left (no pain) to the right (worst possible pain) [33].

### Muscle biopsy

The muscle biopsy procedure was conducted by an experienced physician using a 14-gauge Temno disposable cutting needle (REFT149, CareFusion, Vernon Hills, IL U.S.A) inserted into the vastus lateralis at 3 cm depth and ~20 cm proximal to kneecap. Two biopsied muscle samples were collected at each time point. Local anesthesia (2% lidocaine hydrochloride) was administered prior to the procedure. The baseline biopsy was collected from the right leg, more than 10 days before the exercise challenge. After collection, muscle tissue was immediately placed into 20 ml glass vial containing 10% formalin and then embedded into paraffin wax block. This formalin-fixed paraffin wax-embedded (FFPE) tissues were then cut using serial sectioning protocol and mount on glass slides for staining.

### Immunohistochemistry (IHC) staining

IHC analysis as a semi-quantitative method was conducted by a pathologist at the Taipei Institute of Pathology (Taipei, Taiwan). The XT UltraView DAB v3 and BenchMark XT IHC/ISH Staining Module protocol (Ventana Medical Systems, AR, USA) were used to detect expression of monoclonal antibodies p16^INK4A^ (1:200, ab108349; Cambridge, MA, USA), ß-Gal (1:150, NBP2-45731; Novus Biologicals, USA), and CD34 (Ventana Medical Systems, USA) in muscle serial sections. The binding of primary antibody to a specific antigen was located by enzyme labelled secondary antibodies. The complex was then visualized with hydrogen peroxide substrate and 3,3′-diaminobenzidinetetrahydrochloride (DAB) chromogen, which produced brown precipitates. Pale to dark blue coloration represents cell nuclei, whereas brown coloration represents positively stained antigens.

For p16^INK4a^ detection, slides were deparaffinized, washed twice for 5 min in TBS plus 0.025% Triton X-100 and blocked in 10% normal serum with 1% BSA in TBS for 2 h at room temperature. Slides were drained before applying p16^INK4a^ antibody diluted in TBS with 1% BSA and incubated overnight at 4°C. After two 5-min rinses with TBS 0.025% Triton, the slides were incubated in 0.3% H_2_O_2_ in TBS for 15 min. Enzyme-conjugated anti-rabbit secondary antibody was applied to the slide, diluted in TBS with 1% BSA, and incubated for 1 h at room temperature. After a 5-min rinse in tap water, the slides were counterstained with hematoxylin.

For ß-Gal detection, muscle paraffin sections (2 μm thick) were deparaffinized and rehydrated with xylene and ethanol (100%, 95%, 70% 50% and deionized water). Slides were boiled in 10 mM sodium citrate buffer (pH 6.0), cooled on bench top (30 min), and immersed in distilled water (5 min). Tissue sections were quenched with 3.0% hydrogen peroxidase in methanol for 15 min, washed in distilled water (5 min), washed twice with permeabilization buffer containing 1% animal serum and 0.4% Triton X-100 in Phosphate-buffered saline (PBS-T), and incubated with 5% animal serum in PBS-T for 30 min at room temperature. Primary antibody (diluted in 1% animal serum in PBS) was added, incubated at room temperature for 1–2 h, and overnight incubation at 4°C in a humidified chamber. Sections were washed twice with 1% serum in PBS-T for 10 min before adding anti-mouse secondary antibody to each section. The sections were incubated at room temperature for 1 h before washing twice with 1% serum PBS-T for 10 min each. DAB working solution was prepared and applied to tissue that causes chromogenic reaction. Sections developed brown color with positive reaction. Slides were then immersed in deionized water twice for 2 min then were counterstained with hematoxylin. Pale to dark blue coloration represents cell nuclei, whereas brown coloration represents positively stained antigens.

The samples were washed and then incubated with the anti-rabbit secondary antibody for 10 min. After color development with 3,3′-diaminobenzidine at room temperature for 10 min, the sections were counterstained with hematoxylin for 15 min, dehydrated, and mounted according to the standard protocol.

### Image analysis

The muscle cross-sectional area on the slides were reviewed and captured at 10× magnification (BX50 Olympus microscope, Tokyo, Japan) using MShot Image Analysis System v1.0. The analyses were conducted using manual inspection (Image J, National Institute of Health, USA) of 6 visual fields that contains the most compact and complete muscle fibers (> 50). Positively stained markers were quantified via cells expressing brown coloration as antigen. Positive marker criteria include: (a) muscle cell must be whole and intact; (b) the positive marker must have brown (antigen) stained coloration; (c) the positive marker must be intact/attached with muscle cell; (d) colocalization of positive marker must meet criteria for both markers fulfilling (a), (b), and (c) concurrently. Total positive signals (p16^INK4A+^, CD34^+^, ß-Gal^+^) and colocalization number of positive signals of p16^INK4A+^/CD34^+^ of the images were measured. Positive cell counts were normalized to fibers number.

### Quantitative PCR

RNA was extracted from ~15 mg of biopsied muscle using the RNeasy kit (QIAGEN 74104) after a 60-s homogenization in QIAzol Lysis Reagent (QIAGEN, Hilden, Germany, 79306). One microgram of RNA in a total volume of 20 μl was reverse-transcribed to cDNA using iScript cDNA Synthesis Kit (#170–8890) (Bio-Rad, Hercules, CA, USA). Real-time PCR was performed using CFX Connect Real-Time PCR Detection System (Bio-Rad, Hercules, CA, USA), PrimePCR™ Probe Assay (Bio-Rad, Hercules, CA, USA) and iQ Supermix kit (#170–8862) (Bio-Rad, Hercules, CA, USA). The cycling parameters were: 95°C for 3 min, then 50 cycles at 95°C for 10 s and 58°C for 30 s. Gene expression, normalized to the geometric mean of a housekeeping genes (RPP30), was quantified using the 2−(ΔCt) method and expressed as fold difference relative to the RPP30. The primers and probes used to amplify target were supplied from Bio-Rad PrimePCR™ Probe Assay: p16^INK4a^ (or CDKN2A) (Assay ID: qHsaCEP0057827), CD34 (Assay ID: qHsaCIP0026476), GLB1 (Assay ID: qHsaCEP0057625), MPO (Assay ID: qHsaCEP0049167), and RPP30 (Assay ID: qHsaCEP0052683). Test-retest reliability values for p16^INK4a^ mRNA and MPO mRNA are *r* = 0.87 and *r* = 0.94, respectively.

### Statistical analysis

All results were presented as mean ± standard error (SE). Type 1 error equal or less than 5% for comparing mean difference was considered significant. Two-way ANOVA with repeated measure was used to determine the main effect and interactive effects of time (Pre and Post) and supplement (PLA and Rg1). The % change after exercise from baseline between PLA- and Rg1-trials were analyzed using paired *t*-test. Cohen’s *d* effects sizes were calculated and interpreted as small (*d* = 0.2), medium (*d* = 0.5), and large (*d* = 0.8) [34]. Pearson’s correlation was used to determine the magnitude of association between variables. Correlations were evaluated as follows: trivial (0.00–0.09), small (0.10–0.29), moderate (0.30–0.49), large (0.50–0.69), very large (0.70–0.89), nearly perfect (0.90–0.99), and perfect (1.00) [35].

## References

[1] Boquoi A, Arora S, Chen T, Litwin S, Koh J, Enders GH. Reversible cell cycle inhibition and premature aging features imposed by conditional expression of p16Ink4a. Aging Cell. 2015; 14:139–47. 10.1111/acel.12279 25481981 PMC4326901

[2] Janzen V, Forkert R, Fleming HE, Saito Y, Waring MT, Dombkowski DM, Cheng T, DePinho RA, Sharpless NE, Scadden DT. Stem-cell ageing modified by the cyclin-dependent kinase inhibitor p16INK4a. Nature. 2006; 443:421–26. 10.1038/nature05159 16957735

[3] Justice JN, Gregory H, Tchkonia T, LeBrasseur NK, Kirkland JL, Kritchevsky SB, Nicklas BJ. Cellular Senescence Biomarker p16INK4a+ Cell Burden in Thigh Adipose is Associated With Poor Physical Function in Older Women. J Gerontol A Biol Sci Med Sci. 2018; 73:939–45. 10.1093/gerona/glx134 28658942 PMC6001887

[4] Campisi J. Senescent cells, tumor suppression, and organismal aging: good citizens, bad neighbors. Cell. 2005; 120:513–22. 10.1016/j.cell.2005.02.003 15734683

[5] Spalding KL, Bhardwaj RD, Buchholz BA, Druid H, Frisén J. Retrospective birth dating of cells in humans. Cell. 2005; 122:133–43. 10.1016/j.cell.2005.04.028 16009139

[6] Storer M, Mas A, Robert-Moreno A, Pecoraro M, Ortells MC, Di Giacomo V, Yosef R, Pilpel N, Krizhanovsky V, Sharpe J, Keyes WM. Senescence is a developmental mechanism that contributes to embryonic growth and patterning. Cell. 2013; 155:1119–30. 10.1016/j.cell.2013.10.041 24238961

[7] Yang C, Jiao Y, Wei B, Yang Z, Wu JF, Jensen J, Jean WH, Huang CY, Kuo CH. Aged cells in human skeletal muscle after resistance exercise. Aging (Albany NY). 2018; 10:1356–65. 10.18632/aging.101472 29953414 PMC6046228

[8] Ressler S, Bartkova J, Niederegger H, Bartek J, Scharffetter-Kochanek K, Jansen-Dürr P, Wlaschek M. p16INK4A is a robust *in vivo* biomarker of cellular aging in human skin. Aging Cell. 2006; 5:379–89. 10.1111/j.1474-9726.2006.00231.x 16911562

[9] Dungan CM, Peck BD, Walton RG, Huang Z, Bamman MM, Kern PA, Peterson CA. *In vivo* analysis of γH2AX+ cells in skeletal muscle from aged and obese humans. FASEB J. 2020; 34:7018–35. 10.1096/fj.202000111RR 32246795 PMC7243467

[10] Liu Y, Sanoff HK, Cho H, Burd CE, Torrice C, Ibrahim JG, Thomas NE, Sharpless NE. Expression of p16(INK4a) in peripheral blood T-cells is a biomarker of human aging. Aging Cell. 2009; 8:439–48. 10.1111/j.1474-9726.2009.00489.x 19485966 PMC2752333

[11] Zampetaki A, Kirton JP, Xu Q. Vascular repair by endothelial progenitor cells. Cardiovasc Res. 2008; 78:413–21. 10.1093/cvr/cvn081 18349136

[12] Tamaki T, Akatsuka A, Ando K, Nakamura Y, Matsuzawa H, Hotta T, Roy RR, Edgerton VR. Identification of myogenic-endothelial progenitor cells in the interstitial spaces of skeletal muscle. J Cell Biol. 2002; 157:571–77. 10.1083/jcb.200112106 11994315 PMC2173851

[13] Erben RG, Odörfer KI, Siebenhütter M, Weber K, Rohleder S. Histological assessment of cellular half-life in tissues *in vivo*. Histochem Cell Biol. 2008; 130:1041–46. 10.1007/s00418-008-0470-3 18618128

[14] Ribeiro F, Ribeiro IP, Gonçalves AC, Alves AJ, Melo E, Fernandes R, Costa R, Sarmento-Ribeiro AB, Duarte JA, Carreira IM, Witkowski S, Oliveira J. Effects of resistance exercise on endothelial progenitor cell mobilization in women. Sci Rep. 2017; 7:17880. 10.1038/s41598-017-18156-6 29259281 PMC5736626

[15] Hoffman J. NSCA's Guide to Program Design. Champiagn, IL: Human Kinetics; 2011.

[16] Lasry A, Ben-Neriah Y. Senescence-associated inflammatory responses: aging and cancer perspectives. Trends Immunol. 2015; 36:217–28. 10.1016/j.it.2015.02.009 25801910

[17] Amanzada A, Malik IA, Nischwitz M, Sultan S, Naz N, Ramadori G. Myeloperoxidase and elastase are only expressed by neutrophils in normal and in inflamed liver. Histochem Cell Biol. 2011; 135:305–15. 10.1007/s00418-011-0787-1 21327394 PMC3052504

[18] Kay MM. Mechanism of removal of senescent cells by human macrophages *in situ*. Proc Natl Acad Sci U S A. 1975; 72:3521–25. 10.1073/pnas.72.9.3521 1059140 PMC433027

[19] Lee EJ, Ko E, Lee J, Rho S, Ko S, Shin MK, Min BI, Hong MC, Kim SY, Bae H. Ginsenoside Rg1 enhances CD4(+) T-cell activities and modulates Th1/Th2 differentiation. Int Immunopharmacol. 2004; 4:235–44. 10.1016/j.intimp.2003.12.007 14996415

[20] Zou Y, Tao T, Tian Y, Zhu J, Cao L, Deng X, Li J. Ginsenoside Rg1 improves survival in a murine model of polymicrobial sepsis by suppressing the inflammatory response and apoptosis of lymphocytes. J Surg Res. 2013; 183:760–66. 10.1016/j.jss.2013.01.068 23478085

[21] Wu J, Saovieng S, Cheng IS, Jensen J, Jean WH, Alkhatib A, Kao CL, Huang CY, Kuo CH. Satellite cells depletion in exercising human skeletal muscle is restored by ginseng component Rg1 supplementation. J Funct Foods. 2019; 58:27–33.

[22] Wu J, Saovieng S, Cheng IS, Liu T, Hong S, Lin CY, Su IC, Huang CY, Kuo CH. Ginsenoside Rg1 supplementation clears senescence-associated β-galactosidase in exercising human skeletal muscle. J Ginseng Res. 2019; 43:580–88. 10.1016/j.jgr.2018.06.002 31695564 PMC6823780

[23] Sharpless NE. Ink4a/Arf links senescence and aging. Exp Gerontol. 2004; 39:1751–59. 10.1016/j.exger.2004.06.025 15582292

[24] Chikenji TS, Saito Y, Konari N, Nakano M, Mizue Y, Otani M, Fujimiya M. p16^INK4A^-expressing mesenchymal stromal cells restore the senescence-clearance-regeneration sequence that is impaired in chronic muscle inflammation. EBioMedicine. 2019; 44:86–97. 10.1016/j.ebiom.2019.05.012 31129096 PMC6604166

[25] Tidball JG. Regulation of muscle growth and regeneration by the immune system. Nat Rev Immunol. 2017; 17:165–78. 10.1038/nri.2016.150 28163303 PMC5452982

[26] Vasa M, Fichtlscherer S, Aicher A, Adler K, Urbich C, Martin H, Zeiher AM, Dimmeler S. Number and migratory activity of circulating endothelial progenitor cells inversely correlate with risk factors for coronary artery disease. Circ Res. 2001; 89:E1–7. 10.1161/hh1301.093953 11440984

[27] Fang F, Chen X, Huang T, Lue LF, Luddy JS, Yan SS. Multi-faced neuroprotective effects of Ginsenoside Rg1 in an Alzheimer mouse model. Biochim Biophys Acta. 2012; 1822:286–92. 10.1016/j.bbadis.2011.10.004 22015470 PMC3304026

[28] Wu J, Cheng IS, Saovieng S, Jean WH, Kao CL, Liu YY, Huang CY, Lee TXY, Ivy JL, Kuo CH. Aerobic exercise induces tumor suppressor p16^INK4a^ expression of endothelial progenitor cells in human skeletal muscle. Aging (Albany NY). 2020; 12:20226–34. 10.18632/aging.103763 33104519 PMC7655215

[29] World Medical Association. World Medical Association Declaration of Helsinki: ethical principles for medical research involving human subjects. JAMA. 2013; 310:2191–94. 10.1001/jama.2013.281053 24141714

[30] Moore DR, Churchward-Venne TA, Witard O, Breen L, Burd NA, Tipton KD, Phillips SM. Protein ingestion to stimulate myofibrillar protein synthesis requires greater relative protein intakes in healthy older versus younger men. J Gerontol A Biol Sci Med Sci. 2015; 70:57–62. 10.1093/gerona/glu103 25056502

[31] Helms ER, Cronin J, Storey A, Zourdos MC. Application of the Repetitions in Reserve-Based Rating of Perceived Exertion Scale for Resistance Training. Strength Cond J. 2016; 38:42–49. 10.1519/SSC.0000000000000218 27531969 PMC4961270

[32] Day ML, McGuigan MR, Brice G, Foster C. Monitoring exercise intensity during resistance training using the session RPE scale. J Strength Cond Res. 2004; 18:353–58. 15142026 10.1519/R-13113.1

[33] Sriwatanakul K, Kelvie W, Lasagna L. The quantification of pain: an analysis of words used to describe pain and analgesia in clinical trials. Clin Pharmacol Ther. 1982; 32:143–48. 10.1038/clpt.1982.139 7094502

[34] Cohen J. Statistical power analysis for the behavioral sciences. Academic press. 2013.

[35] Hopkins WG. A scale of magnitudes for effect statistics. A new view of statistics. 2002. https://www.sportsci.org/resource/stats/effectmag.html

